# Questionable Operations

**Published:** 1880-10

**Authors:** N. F. Thompson

**Affiliations:** New York


### "QUESTIONABLE OPERATIONS."

By N. F. Thompson, D.V.S.

IN the August number of the American *Veterinary Review* for 1880, page 212, we notice an article entitled "Questionable Operations?" citing two out of four cases reported in the "Archives of Comparative Medicine and Surgery" for July, 1880.

The author of the article questions the diagnosis, also the advisability of operative interference, and intimates that it is unnecessary in operating to remove so much underlying muscular tissue, that the animal is in some degree disabled. The author also advocates the older course of treatment, such as powerful caustics, or the seaton, as the only course to insure complete removal without injury to the muscular tissue. This plan of treatment was familiar to both operator and author; was tried and failed, in one of the cases where operative interference afterwards proved successful.

As there seems to be a doubt in the minds of some regarding the character of these growths, it may be well to state that they were either purely fibrous, or fibrous interspersed with a little adipose tissue; not one of these tumors even contained a pocket of pus in the centre, neither was there anything about them to lead one to think that they had orignated as abscesses, and that they were chronic abscesses with thickened walls.

If it became necessary, in the removal of these tumors, to remove so much of the underlying muscular tissue that the animal became in any degree disabled by muscular contractions; and if it is a fact that the growths can be completely removed by caustics or seatons, without disabling the animal, the latter would be preferable, even though it took more time to attain the final result; but if operative interference is equally as successful and more speedy, then it should be resorted to in all cases.

In the four cases reported in the ARCHIVES not a single animal was disabled by the removal of muscular tissue; in every case the result was perfect and more speedy than that of the caustic or seaton. Is also a well-known fact that large ulcerating surfaces are apt to leave large and hard cicatrices, which if at a point of constant irritation, would very likely be followed by a return of the indurated mass, while the wound of the operation would be less likely to result in a large, hard cicatrix.

Even if these tumors were found to be chronic abscesses, operative interference would be justifiable, and ought to be resorted too, for the simple reason that recovery would in all probability be more speedy, and equally if not more successful.

For the above reasons we believe that operative interference is justifiable, and ought to be resorted to for the removal of such tumors, and also chronic abscesses, as it is more scientific, and shows an advance in Veterinary Surgery.

New York September 25th, 1880.

